# Ultrasound Thawing Enhances Myofibrillar Protein Integrity and Quality Attributes of Common Carp (*Cyprinus carpio*) Fillets

**DOI:** 10.1002/fsn3.71833

**Published:** 2026-05-18

**Authors:** Mohammad Mehdi Asgari, Mina Esmaeili, Abbas Zamani, Seyed Vali Hosseini

**Affiliations:** ^1^ Department of Fisheries, Faculty of Animal Sciences and Fisheries Sari Agricultural Sciences and Natural Resources University Sari Iran; ^2^ Department of Fisheries, Faculty of Natural Resources and Environment Malayer University Malayer Iran; ^3^ Department of Fisheries, College of Agriculture & Natural Resources University of Tehran Karaj Alborz Iran

**Keywords:** common carp ( *Cyprinus carpio* ), myofibrillar protein, quality attributes, seafood technology, thawing methods, ultrasound

## Abstract

Freezing and thawing are critical techniques for preserving seafood quality and extending shelf life. This study evaluated the effects of ultrasound‐assisted (UT), water immersion (WT), and microwave (MT) thawing on physicochemical, textural, and structural quality attributes of common carp ( *Cyprinus carpio* ) fillets and their myofibrillar protein properties. Results demonstrated that ultrasound thawing (UT) most effectively maintained pH and minimized protein aggregation, reducing myofibrillar protein particle size by approximately 49%, significantly lowering turbidity, and decreasing cooking loss by about 10% compared with water immersion thawing (WT). In contrast, water immersion thawing resulted in the greatest moisture loss, whereas ultrasound and microwave thawing better preserved water‐holding capacity. Scanning electron microscopy revealed that WT and MT caused minor muscle damage, whereas UT better preserved protein integrity, showing significantly reduced aggregation and higher zeta potential compared with MT (*p* < 0.05). SDS‐PAGE confirmed that no treatments altered the primary protein structure. Based on overall quality retention, ultrasound thawing is recommended as the optimal thawing method for common carp fillets.

## Graphical abstract

Effects of different thawing methods on protein integrity and quality of common carp fillets.

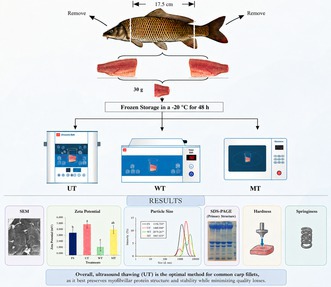

## 1 Introduction

Common carp ( *Cyprinus carpio* ) is a commercially important warm‐water fish species belonging to the Cyprinidae family. Owing to its rapid growth rate, adaptability to cultivation, and efficient feed conversion ratio, it is extensively farmed worldwide (Tokur et al. 2006). According to the Food and Agriculture Organization (FAO), this species accounts for approximately 8% of global aquaculture production, yielding around 2.4 million tons annually and ranking as the fourth most cultured fish globally (FAO 2022). Despite its nutritional value, common carp is highly perishable at ambient temperatures due to its elevated water activity. Common carp was selected as the model species due to its high global production and economic importance in freshwater aquaculture. Moreover, as a widely marketed species in both fresh and frozen forms, it represents a commercially relevant model for evaluating the impact of thawing technologies on muscle quality and protein stability. To mitigate spoilage, the food industry employs various preservation techniques, including drying, refrigeration, and freezing, with freezing being the most effective method for maintaining quality and prolonging shelf life (Li et al. 2018). Freezing slows metabolic reactions that lead to undesirable textural and color changes, nutrient degradation, and the development of off‐flavors and odors (e.g., protein denaturation and lipid oxidation) (Cheng et al. 2015).

However, after freezing, the thawing process plays an equally crucial role in preserving fish quality, as the chosen method and conditions directly influence texture and nutritional integrity. Thawing is an essential preparatory step for subsequent processing, such as consumption, cutting, or further preparation (Wen et al. 2015). Nevertheless, improper thawing can adversely affect muscle quality. Key factors determining the extent of thawing‐induced changes include duration, temperature, and the specific thawing technique employed (Choi et al. 2017; Qian et al. 2019).

Thawing involves the phase transition of frozen water back to liquid, a process requiring substantial energy input and typically occurring between −5°C and 0°C. Due to the high energy demand, products often remain within this critical temperature range for prolonged periods, increasing the risk of quality deterioration, such as excessive drip loss. Thus, rapid and controlled thawing is essential to minimize damage, underscoring the importance of selecting an appropriate thawing method (Ragnarsson and Viðarsson 2017).

Traditional thawing techniques, such as water immersion (Choi et al. 2017), refrigerator storage (Qian et al. 2019), and air thawing (Li et al. 2019), rely on external heat transfer, which is time‐consuming and often compromises product quality (James et al. 2017). In contrast, modern methods, including microwave (Choi et al. 2017) and ultrasonic thawing (Zou et al. 2019) generate heat internally, ensuring more uniform thawing (Cai, Cao, et al. 2019; Schubring et al. 2003). These methods differ significantly in their effects on biochemical and physical properties, including flavor (Jia et al. 2019), texture (James et al. 2017), color (Alvarenga et al. 2019), protein stability (Cai et al. 2018), and functional protein characteristics (Jia et al. 2018).

Water immersion thawing is currently one of the most common traditional methods used by commercial companies for large‐scale thawing. Immersing fish blocks for several hours in water tanks with no or low water flow may require minimal technology and knowledge but can have many adverse effects on the quality of the final product. In this thawing method, water circulation is not properly conducted, and there is generally no water purification and filtration system. As a result, contaminants and bacteria present in the gastrointestinal system and blood can easily transfer from one fish to another, contaminating the entire water tank. Furthermore, inadequate temperature regulation may either prolong thawing times (if too low) or cause overheating (if too high), both of which degrade product quality (Ragnarsson and Viðarsson 2017).

Microwave thawing offers a rapid, energy‐efficient, and hygienic alternative, suitable for both industrial and household applications. By generating heat internally, it avoids excessive surface heating, ensuring faster and more uniform thawing (Cai et al. 2018; Cao et al. 2018). Similarly, ultrasound‐assisted thawing has recently gained attention for improving thawing efficiency and preserving muscle quality. Recent studies demonstrated that appropriate ultrasound power can inhibit myofibrillar protein oxidation and aggregation, enhance gel strength and water‐holding capacity in common carp myofibrillar protein during thawing (Sun et al. 2024), and moderate ultrasound treatments reduced structural changes and emulsifying property loss in fish proteins (Sun et al. 2023). Ultrasound thawing also shortened thawing time and improved texture and water retention in meat products (Sun et al. 2023), and optimization of ultrasound parameters has been shown to limit protein oxidation and quality deterioration in poultry (Santos et al. 2025). Furthermore, reviews on ultrasound applications in aquatic protein processing highlight its potential to protect protein structure and functionality during freeze–thaw processes (Zheng et al. 2024).

Despite increasing interest in ultrasound‐assisted thawing, limited is essential studies have provided an integrated evaluation of physicochemical, textural, microstructural, and myofibrillar protein properties in common carp. Given the commercial importance of this species, such comprehensive assessment. Therefore, this study aims to systematically compare ultrasound, microwave, and water immersion thawing methods, with particular emphasis on protein stability, structural integrity, and overall quality attributes.

## 2 Materials and Methods

### 2.1 Sample Preparation

#### 2.1.1 Materials

Sample preparation followed the methodology of Sun et al. (2019) with minor modifications (Sun et al. 2019). Ten common carp, averaging 1.1–1.3 kg in weight and approximately 34 cm in length, were obtained from a local aquaculture facility in Karaj, Iran. The live fish were transported to the University of Tehran's Processing Laboratory within 30 min in temperature‐controlled water (25°C). Euthanasia was performed by administering one or two precise strikes to the cephalic region using a metal rod. Following euthanasia, specimens were thoroughly rinsed with cold water, then subjected to beheading, evisceration, and skin removal.

**FIGURE 1:**
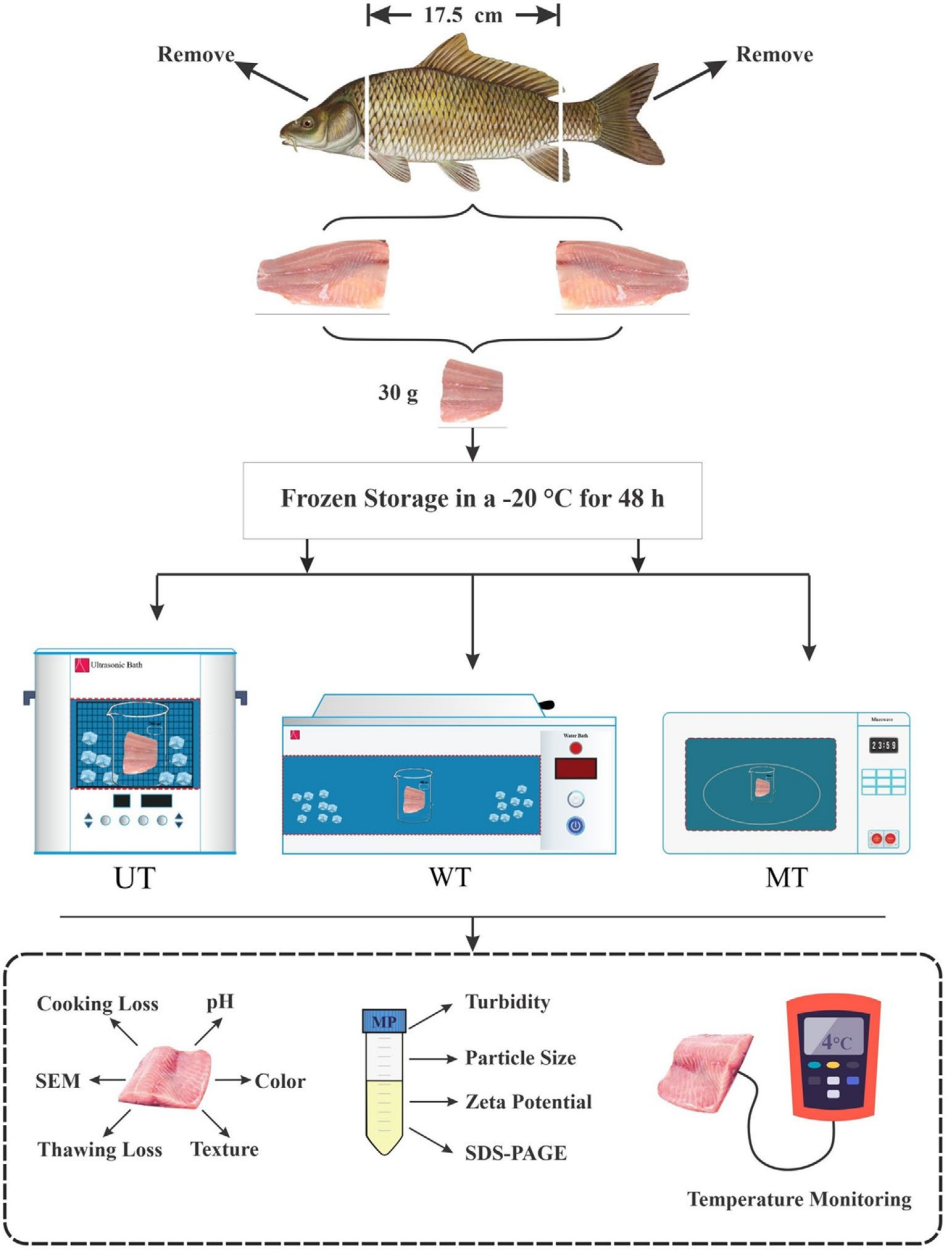
Schematic representation of experimental treatments: UT (ultrasound thawing), WT (water immersion thawing), MT (microwave thawing), and MP (myofibrillar protein).

As shown in Figure 1, two fillets were obtained from the dorsal region of each fish and then portioned into uniform 30 ± 0.1 g portions, randomly packaged, and frozen without tracking individual fish origin. Polyethylene bags were used to individually package each sample to prevent moisture loss during storage. The packaged samples were subsequently frozen at −20°C for 48 h to ensure complete freezing. For each analysis, one 30 g portion was used as the experimental unit and subdivided into three 10 g subsamples for triplicate measurements. Therefore, replicate measurements represent technical replicates performed on subsamples of the same experimental unit. The experimental treatments were randomly categorized as follows: (1) Fresh Samples (FS), (2) Ultrasound Thawing (UT), (3) Water Immersion Thawing (WT), and (4) Microwave Thawing (MT). All experimental procedures were conducted in triplicate to ensure statistical reliability.

#### 2.1.2 Thawing Methods

All thawing procedures were conducted until samples reached a core temperature of 4°C (±0.1°C), monitored using a type K thermocouple (1 mm diameter) inserted at the geometric center of each fillet. After thawing, samples were immediately transferred to refrigerated storage at 4°C for subsequent analysis.

##### 2.1.2.1 Ultrasound Thawing (UT)

Ultrasound‐assisted thawing was performed using an ultrasonic bath operating at a frequency of 40 kHz and the power was set at 200 W (Backer Co., Iran; 200 W, 40 kHz, 9 L capacity). Fillets were placed in a 500 mL beaker containing 250 mL deionized water, which was then positioned in the ultrasonic chamber filled with 5 L of deionized water. The system maintained a constant water temperature of 15°C through ice supplementation according to the temperature monitor of ultrasonic bath (Cai et al. 2018).

##### 2.1.2.2 Water Immersion Thawing (WT)

The water immersion thawing protocol maintained identical sample placement conditions to the ultrasound method. Samples were thawed in a temperature‐controlled water bath (Shimaz, Iran; 10 L capacity) with continuous temperature regulation. The bath temperature was maintained at 14.0°C ± 0.5°C through periodic ice supplementation, monitored using the thermometer (Wang et al. 2020).

##### 2.1.2.3 Microwave Thawing (MT)

Microwave‐assisted thawing was conducted using a microwave oven (Samsung Gen 281, Malaysia) operating at 2450 MHz with 300 W output power (Cai, Wan, et al. 2020). Frozen fillet samples were placed in 250 mL glass beakers and positioned at the center of the microwave cavity. The thawing process was monitored until samples reached the target core temperature.

### 2.2 pH Measurement

Sample pH was determined following the method of Li et al. (2020) with modifications. For each measurement, 2.5 g of fresh or thawed fish tissue was homogenized in 25 mL distilled water using a homogenizer (IKA T25D, Germany) at 8000 rpm for 60 s. The homogenate was allowed to equilibrate for 30 min at ambient temperature before pH measurement using a calibrated benchtop pH meter (Shimifann PL‐700, Iran).

### 2.3 Thawing Loss Quantification

Thawing loss was determined gravimetrically according to Hong et al. (2013). All samples were weighed (*W*_0_) and labeled prior to freezing. Following thawing, samples were reweighed (*W*_1_). Thawing loss percentage was calculated as:

$$\text{Thawing loss}\;\left(\%\right)=\left[\left(W_{0}–W_{1}\right)/W_{0}\right]\times 100$$

### 2.4 Cooking Loss Determination

Cooking loss was evaluated following Hong et al. (2013) with modifications. Approximately 5 g of thawed fillet (*W*_a_) from each treatment placed in a glass beaker. The beaker containing the sample was weighed (*W*_b_) and then cooked in a temperature‐controlled water bath at 80.0°C ± 0.5°C for precisely 30 min. After cooking, the beaker and sample were reweighed (*W*_c_). Cooking loss percentage was calculated as:

$$\text{Cooking loss}\;\left(\%\right)=\left[\left(W_{b}–W_{c}\right)/W_{a}\right]\times 100$$

### 2.5 Color Analysis

Surface color parameters were measured following Li et al. (2020), using a colorimeter (CR‐400, Konica Minolta, Tokyo, Japan) to assess the color indices a* (redness/greenness), b* (blueness/yellowness), and L* (lightness/darkness) of fresh and thawed samples arranged on transparent plates. Total color difference (Δ*E*) was calculated as:

$$\Delta E={\left[{\left(\Delta L^{*}\right)}^{2}+{\left(\Delta a^{*}\right)}^{2}+{\left(\Delta b^{*}\right)}^{2}\right]}^{1/2}$$

### 2.6 Texture Profile Analysis (TPA)

Texture properties were evaluated using a texture analyzer (CT3 10K, Brookfield, USA) following Gaarder et al. (2012) with modifications. Fresh and thawed samples were cut into 1.5 × 1.5 × 1 cm cubes. A cylindrical probe (TA4/1000; 38.1 mm diameter × 20 mm length) was used to compress the samples by 50% relative to their height in two compression cycles. Measurements were performed with a pre‐test speed of 3 mm/s, a test speed of 1 mm/s, and a post‐test speed of 1 mm/s. Three independent sample pieces were used for each measurement, which was done three times. Three independent sample pieces were analyzed per treatment, with three replicate measurements performed for each piece.

### 2.7 Scanning Electron Microscopy (SEM)

Glutaraldehyde solution was used to fix slices of both fresh and thawed fish samples for 24 h at 4°C. Samples were then treated with osmium acid for 1–2 h and rinsed three times with phosphate buffer. After dehydration through a series of ethanol solutions, drying, and gold coating, samples were imaged using a scanning electron microscope (FES‐SEM, MIRA3, Tescan Co., Czech Republic), operating at an accelerating voltage of 20 kV and a magnification of 500× (Li et al. 2020).

### 2.8 Myofibrillar Protein (MP) Extraction and Quantification

Myofibrillar protein was extracted according to Cai, Zhang, et al. (2019) with minor adjustments. Minced fish muscle was homogenized (T25D, IKA, Germany) in Buffer A (20 mM NaPO_4_, 20 mM NaCl, 1 mM EDTA, pH 7.0) at a 5:1 (w/v) ratio for 60 s in an ice bath. The homogenate was centrifuged (RFT‐15000‐REFRI, Partazma, Iran) at 6000 rpm for 15 min at 4°C. After discarding the supernatant, the pellet was resuspended in Buffer A (4:1 w/v) and centrifuged again under identical conditions. The resulting pellet was then dissolved in Buffer B (25 mM NaPO_4_, 0.6 M NaCl, pH 7.0) at a 4:1 (w/v) ratio, homogenized, and centrifuged. The final supernatant containing myofibrillar proteins was collected for analysis. Protein concentration was determined using the Biuret method (Gornall et al. 1949). Absorbance measurements were taken at 540 nm using a spectrophotometer (S 2100 SUV, Planfield, USA).

#### 2.8.1 Sodium Dodecyl Sulfate‐Polyacrylamide Gel Electrophoresis (SDS‐PAGE)

Samples were mixed with loading buffer and heat‐denatured at 95°C for 5 min. Aliquots (10 μL) were loaded onto 12% separating gels. Electrophoresis was initiated at 80 V through the stacking gel, then increased to 120 V for the separating phase. According to Laemmli (1970) and Li et al. (2020), the gel was stained with Coomassie Brilliant Blue R‐250 for 30 min and destained in methanol: acetic acid: water (30:10:60 v/v/v) until clear protein bands were visible.

#### 2.8.2 Particle Size Distribution and Zeta Potential

The particle size distribution (PSD) and zeta potential of myofibrillar protein solutions (0.5 mg/mL concentration) were measured using a dynamic light scattering (DLS) system (SZ100 zeta nano‐sizer, Horiba Ltd., Japan). For PSD analysis, protein solutions were loaded into 1 cm path length cylindrical cuvettes and measured at room temperature with a 90° detection angle. Zeta potential measurements were conducted under identical temperature conditions following established protocols (Dara et al. 2021).

#### 2.8.3 Turbidity Measurement

Turbidity of myofibrillar protein solutions was determined following Zhang et al. (2021) methods was used to assess the turbidity of the myofibrillar protein samples. Samples (0.5 mg/mL in 50 mM phosphate buffer containing 0.6 M KCl, pH 7.0) were analyzed using a spectrophotometer (S 2100 SUV, Planfield, USA) at 660 nm absorbance.

### 2.9 Statistical Analysis

Data analysis was performed using SPSS 22 software in a completely randomized design. Significant differences among thawing methods were identified by one‐way ANOVA (*p* < 0.05) followed by Duncan's multiple range test. All experiments were conducted in triplicate and results were plotted using Microsoft Excel.

## 3 Results and Discussion

### 3.1 Changes in Tissue Structure

#### 3.1.1 pH, Thawing Loss and Cooking Loss

The effects of thawing methods on pH, thawing loss, and cooking loss in common carp fillets are presented in Table 1. pH serves as a critical indicator of fish quality. Results indicated a decrease in pH from 7.08 to 6.86 across treatments, demonstrating that thawing methods significantly influence pH. While ultrasound‐thawed (UT) samples showed no significant difference from fresh samples (FS) or other methods, water immersion (WT) and microwave thawing (MT) differed significantly from the control (*p* < 0.05). Gambuteanu and Alexe (2015) reported that ultrasound treatment did not cause pH alteration during thawing process and the slight decrease in pH may be attributed to post‐mortem anaerobic metabolism, during which muscle glycogen is converted into lactic acid, leading to acidification of frozen fish tissue (Liu et al. 2013). This aligns with Li et al. (2020), who reported slight pH changes (*p* > 0.05) were observed between WIT and UAT bighead carp (*Aristichthys nobilis*) samples.

**TABLE 1:** pH, thawing loss, and cooking loss of common carp ( *Cyprinus carpio* ) fillets subjected to different thawing treatments.

Treatment	pH	Thawing loss (%)	Cocking loss (%)
FS	7.08 ± 0.09^a^	—	15.55 ± 0.02^c^
UT	6.94 ± 0.02^ab^	1.49 ± 0.43^a^	18.95 ± 0.16^b^
WT	6.90 ± 0.02^b^	1.06 ± 0.55^a^	21.01 ± 0.08^a^
MT	6.86 ± 0.02^b^	1.18 ± 0.22^a^	19.61 ± 0.15^b^

*Note:* Values are mean ± SD (*n* = 3). Different superscript letters within a column indicate significant differences (*p* < 0.05).

Abbreviations: FS, fresh sample; MT, microwave thawing; UT, ultrasound thawing; WT, water immersion thawing.

Thawing loss, a key factor affecting product quality and economic value (Hong et al. 2013), was lowest in WT, followed by MT and UT, though differences were non‐significant. This loss primarily results from ice crystal formation during freezing and subsequent tissue damage (Wu et al. 2017). Li et al. (2020) similarly observed no significant differences in thawing loss among methods for bighead carp, noting that water‐holding capacity remained largely unaffected.

Cooking loss differed significantly (*p* < 0.05) between all thawed samples and FS (Table 1). WT exhibited the highest cooking loss (21.01 ± 0.08), significantly exceeding UT and MT (*p* < 0.05). No significant difference occurred between UT and MT (*p* > 0.05). Cooking loss arises from fluid leakage and soluble compound loss due to structural damage from myofibrillar protein denaturation under thermal stress (Li et al. 2020; Sun et al. 2019). Ice crystal formation and melting during freeze–thaw cycles weaken cell adhesion and damage cellular integrity, facilitating moisture escape during heating. This reduced water retention directly impairs textural properties (hardness, chewiness, elasticity) and depletes water‐soluble nutrients (Oz and Zikirov 2015). These findings align with Cai, Wan, et al. (2020) who reported significantly higher cooking loss in thawed largemouth bass ( *Micropterus salmoides* ) versus fresh controls across all methods.

#### 3.1.2 Color Evaluation

Fillet color is a critical factor influencing consumer acceptance, directly linked to water distribution within tissues (Hughes et al. 2014) and serving as a primary visual freshness indicator (Li et al. 2020). Table 2 presents the impact of thawing methods on color parameters. After thawing, microwave‐treated (MT) samples showed significantly higher L* values than fresh samples (FS) (*p* < 0.05). Ultrasound (UT) and water immersion (WT) showed no significant L* difference, consistent with Li et al. (2020), who reported L* values of 58.66 ± 0.28 for WT and 58.31 ± 0.43 for UT, as well as with the findings of Xu et al. (2021). No significant differences in a* values occurred between FS and thawed groups which aligns with the findings of Li et al. (2020). UT showed significantly higher b* than FS (*p* < 0.05), possibly reflecting oxidation from ice crystal damage (Li et al. 2020; Sun et al. 2021). Although slight variations in ΔE were observed, all values remained below the commonly accepted perceptibility threshold (~2–3), indicating that these differences are unlikely to be noticeable to consumers.

**TABLE 2:** Color parameters (L*, a*, b*, Δ*E*) of common carp ( *Cyprinus carpio* ) fillets under different thawing treatments.

Treatment	∆*E*	L*	a*	b*
FS	61.04 ± 2.36^a^	36.41 ± 2.56^b^	7.13 ± 1.32^a^	20.22 ± 0.61^b^
UT	59.89 ± 2.63^a^	38.70 ± 2.50^ab^	6.92 ± 1.29^a^	22.99 ± 0.51^a^
WT	60.59 ± 1.62^a^	37.51 ± 1.53^ab^	8.41 ± 0.98^a^	21.52 ± 2.73^ab^
MT	58.15 ± 1.59^a^	39.75 ± 1.59^a^	6.96 ± 1.29^a^	20.99 ± 1.14^b^

*Note:* Values are mean ± SD (*n* = 3). Different superscript letters within a column indicate significant differences (*p* < 0.05).

Abbreviations: FS, fresh sample; MT, microwave thawing; UT, ultrasound thawing; WT, water immersion thawing.

#### 3.1.3 Texture Evaluation

Texture serves as a valuable indicator of the sensory and functional properties of fish (Liu et al. 2014). As presented in Table 3, this study evaluated texture properties through several parameters: hardness, chewiness, resilience, cohesiveness, springiness, and gumminess.

**TABLE 3:** Texture profile analysis of common carp ( *Cyprinus carpio* ) fillets under different thawing treatments.

Treatment	Hardness (g)	Chewiness (mj)	Resilience	Cohesiveness	Springiness	Gumminess
FS	5714.4 ± 238.4^a^	47.34 ± 7.29^a^	0.144 ± 0.00^a^	0.202 ± 0.02^a^	4.512 ± 0.3^a^	1066.8 ± 109.36^a^
UT	3117.8 ± 840.06^c^	13.4 ± 4.13^b^	0.114 ± 0.03^ab^	0.164 ± 0.03^b^	2.7 ± 0.12^c^	506.8 ± 161.92^c^
WT	4309.6 ± 600.23^b^	17.64 ± 2.93^b^	0.138 ± 0.03^a^	0.174 ± 0.01^ab^	2.426 ± 0.14^c^	746.2 ± 152.07^b^
MT	3920 ± 765.99^bc^	19.64 ± 5.47^b^	0.088 ± 0.00^b^	0.148 ± 0.01^b^	3.404 ± 032^b^	581.2 ± 119.04^bc^

*Note:* Values represent mean ± standard deviation (*n* = 3). Different superscript letters within a column indicate significant differences (*p* < 0.05).

Abbreviations: FS, fresh sample; MT, microwave thawing; UT, ultrasound thawing; WT, water immersion thawing.

Hardness represents the most critical factor in assessing fish texture. All thawed samples exhibited significantly lower hardness than the control (*p* < 0.05). Among treatments, water immersion‐thawed (WT) samples demonstrated significantly higher hardness than ultrasound‐thawed (UT) samples (*p* < 0.05), though no significant difference was observed between WT and microwave‐thawed (MT) samples (*p* > 0.05). Additionally, all three treatments showed significantly lower chewiness values compared to the control sample (*p* < 0.05). Regarding resilience, UT and MT treatments displayed lower values than control samples, with MT showing a significant decrease (*p* < 0.05). WT results resembled control values with no significant difference (*p* > 0.05). These findings align with Li et al. (2020), who reported similar reductions in hardness, chewiness, and resilience for water and ultrasound‐thawed bighead carp (*Aristichthys nobilis*) under various freezing conditions.

In terms of cohesiveness, all treatments showed lower values than control, with significant decreases observed in UT and MT samples (*p* < 0.05), consistent with Cai, Wan, et al. (2020). For springiness, all samples exhibited significantly lower values than control (*p* < 0.05), though MT samples most closely approximated control values, indicating better springiness retention. This finding is in line with Cai, Wan, et al. (2020).

Finally, control samples demonstrated significantly higher gumminess than all treatments (*p* < 0.05). Among treatments, WT showed significantly higher gumminess than UT (*p* < 0.05), while no significant difference was found between WT and MT (*p* > 0.05). This correlation between gumminess and hardness has been previously documented (Cai, Dai, and Cao 2020). The observed texture changes may be attributed to tissue denaturation resulting from various factors, including water loss from the muscles and tissue degradation (Liu et al. 2013). The lower values obtained from the different thawing methods compared to the control sample may be due to reduced water‐holding capacity and tissue degradation. Overall, the treatments can be ranked as follows: FS > WT > MT > UT.

#### 3.1.4 Scanning Electron Microscopy (SEM)

**FIGURE 2:**
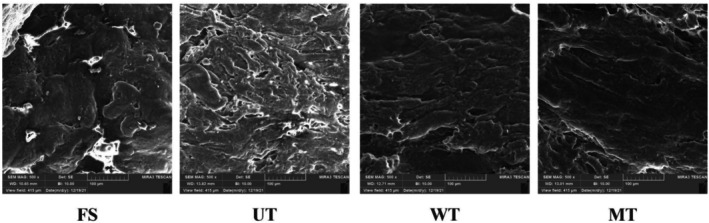
Scanning electron micrographs of common carp ( *Cyprinus carpio* ) fillets subjected to different thawing treatments. FS, fresh sample; MT, microwave thawing; UT, ultrasound thawing; WT, water immersion thawing. Scale bars: 50 μm.

Microstructural architecture serves as a critical indicator of muscle tissue integrity (Sun et al. 2021). Scanning electron microscopy (SEM) revealed structural differences among treatments (Figure 2). Fresh samples exhibited smooth, continuous muscle fibers with minimal curvature or breakage. In contrast, thawed samples showed varying degrees of structural disruption, including cavities and surface irregularities.

Among thawing treatments, water immersion‐thawed (WT) and microwave‐thawed (MT) samples most closely resembled FS, indicating relatively preserved microstructure. These observations align with texture data (Table 3), where WT and MT maintained higher hardness values. The structural gaps in treated samples likely resulted from ice crystal formation and mechanical disruption during freezing/thawing (Wu et al. 2017).

In ultrasound‐thawed samples, increased inter‐fiber gaps were observed compared to other treatments. Howeve, thisdoes not necessarily indicate structural deterioration. Instead, it may result from localized cavitation effects, which induce micro‐level disruption and promote structural loosening, thereby enhancing mass transfer during thawing. Importantly, protein‐level analyses (Section 3.2) demonstrated reduced aggregation, as reflected by smaller particle size, lower turbidity, and higher zeta potential. These findings indicate that microstructural loosening and improved protein stability can coexist, suggesting that ultrasound‐induced structural modifications are not detrimental but functionally beneficial.

### 3.2 Alterations in the Structure of Myofibrillar Proteins

Aggregation and degradation of myofibrillar proteins indicate changes in protein composition (Cao et al. 2018), which can be evaluated through SDS‐PAGE, particle size analysis, and zeta potential. Electrophoretic band patterns reveal the extent of protein aggregation, cross‐linking, and changes in chemical bonds (Cai, Zhang, et al. 2019). An increase in particle size may suggest the formation of aggregates or the degradation of hydrogen bonds within the protein structure. A higher zeta potential value generally indicates greater structural stability of the myofibrillar proteins (Cai et al. 2018).

#### 3.2.1 SDS‐Page

**FIGURE 3:**
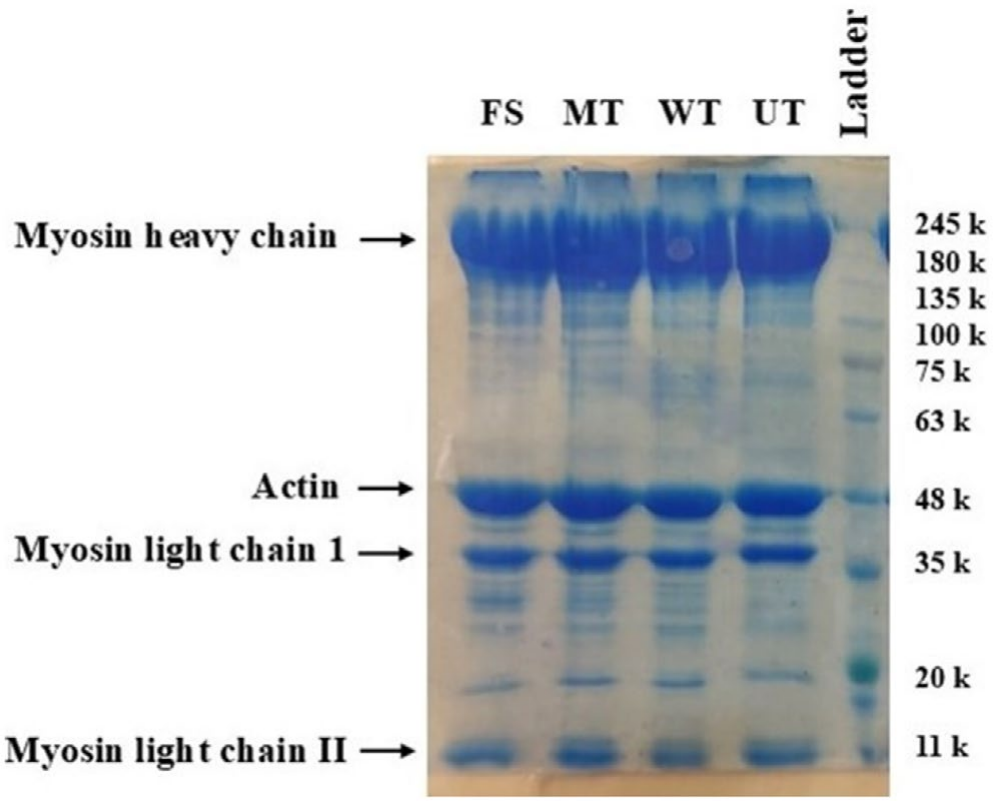
SDS‐Page analysis of myofibrillar proteins from common carp ( *Cyprinus carpio* ) fillets subjected to different thawing treatments. FS, fresh sample; MT, microwave thawing; UT, ultrasound thawing; WT, water immersion thawing.

As shown in Figure 3, The bands corresponding to actin (~48 kDa) and heavy myosin chains (MHC, ~200 kDa) were more intense than others. The presence of high molecular weight bands indicates cross‐linking and aggregation, while the appearance of lower molecular weight bands suggests the degradation of heavy chains into lighter fragments. According to Cheng et al. (2017), this degradation may be attributed to the cold denaturation of muscle proteins, which refers to structural instability caused by changes in hydrophobic interactions, hydrogen bonding, and water molecule density on the protein surface at temperatures below −18°C (Van Dijk et al. 2016). Although strong covalent bonds help maintain the primary structure of proteins, cold denaturation typically occurs without disrupting these bonds. Therefore, freezing generally does not significantly alter the protein's primary structure (Li et al. 2020). In the present study, ultrasound, microwave, and water immersion thawing did not affect the primary structure of tissue proteins, which is consistent with the findings of Li et al. (2020), who reported similar results for Chinese shrimp ( *Fenneropenaeus chinensis* ) under ultrasound thawing.

#### 3.2.2 Protein Aggregation

**FIGURE 4:**
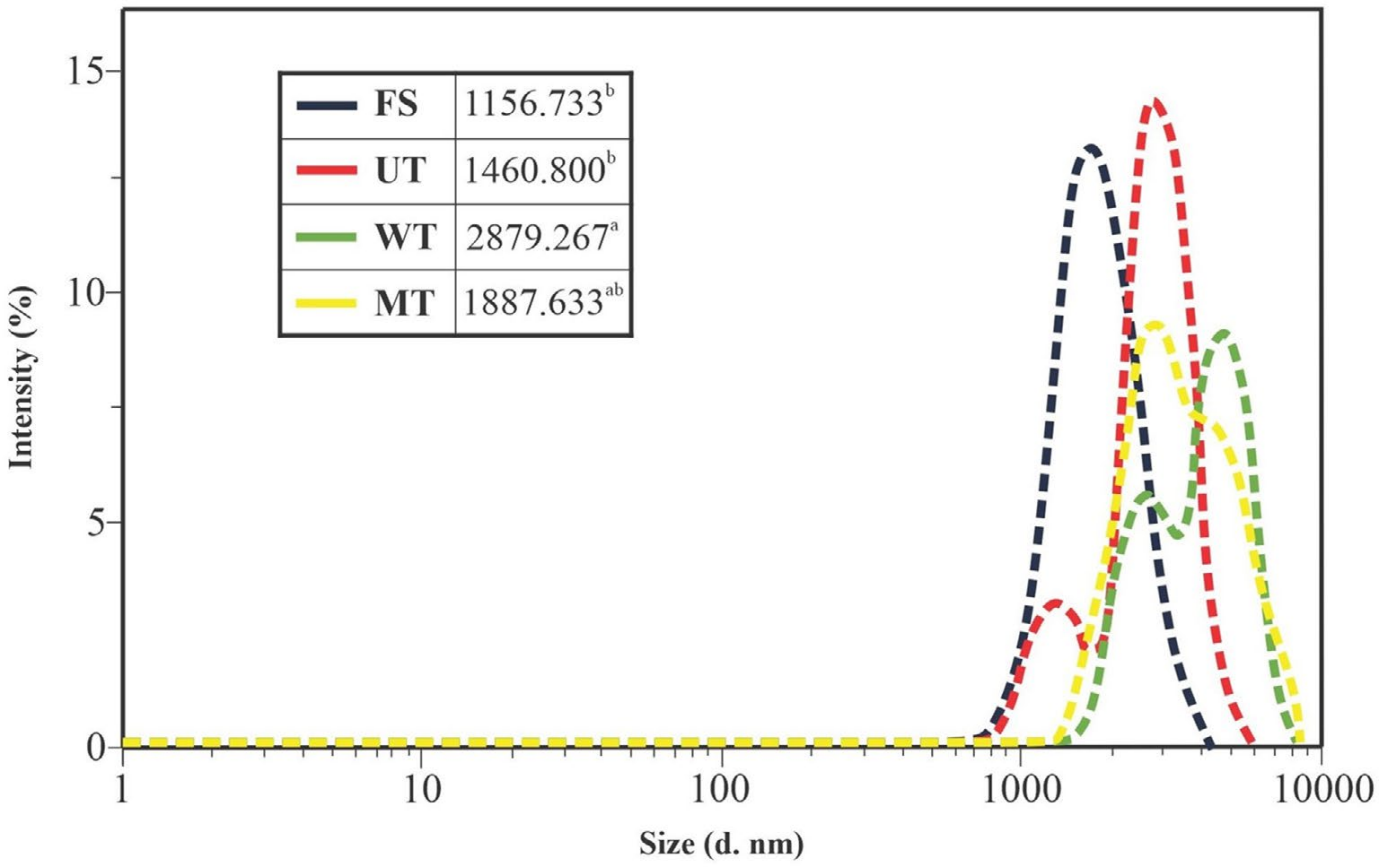
Particle size distribution of myofibrillar proteins from common carp ( *Cyprinus carpio* ) fillets under different thawing treatments. FS, fresh sample; MT, microwave thawing; UT, ultrasound thawing; WT, water immersion thawing.

Particle size of soluble proteins serves as a key indicator of protein aggregation extent (Cao et al. 2019). As shown in Figure 4, the average particle sizes of myofibrillar proteins following UT, WT, and MT treatments were 1460.80, 2879.26, and 1887.63 nm, respectively. Oxidation and denaturation during processing and storage can promote myofibrillar protein aggregation, leading to increased particle size (Cao and Xiong 2015).

Among the thawing treatments, ultrasound (UT) yielded the smallest particle size. The observed reduction in particle size in UT samples can be attributed to acoustic cavitation. The collapse of microbubbles generates localized shear forces that disrupt protein aggregates and enhance dispersion stability. In addition, microstreaming enhances mass transfer, reducing the likelihood of protein re‐aggregation (Yanjun et al. 2014). These findings align with Cai et al. (2018), who reported minimal particle size changes in ultrasound (0.91 ± 0.11) and microwave‐thawed (1.0 ± 0.1) red sea bream ( *Pagrus major* ) compared to fresh controls (0.91 ± 0.07).

**FIGURE 5:**
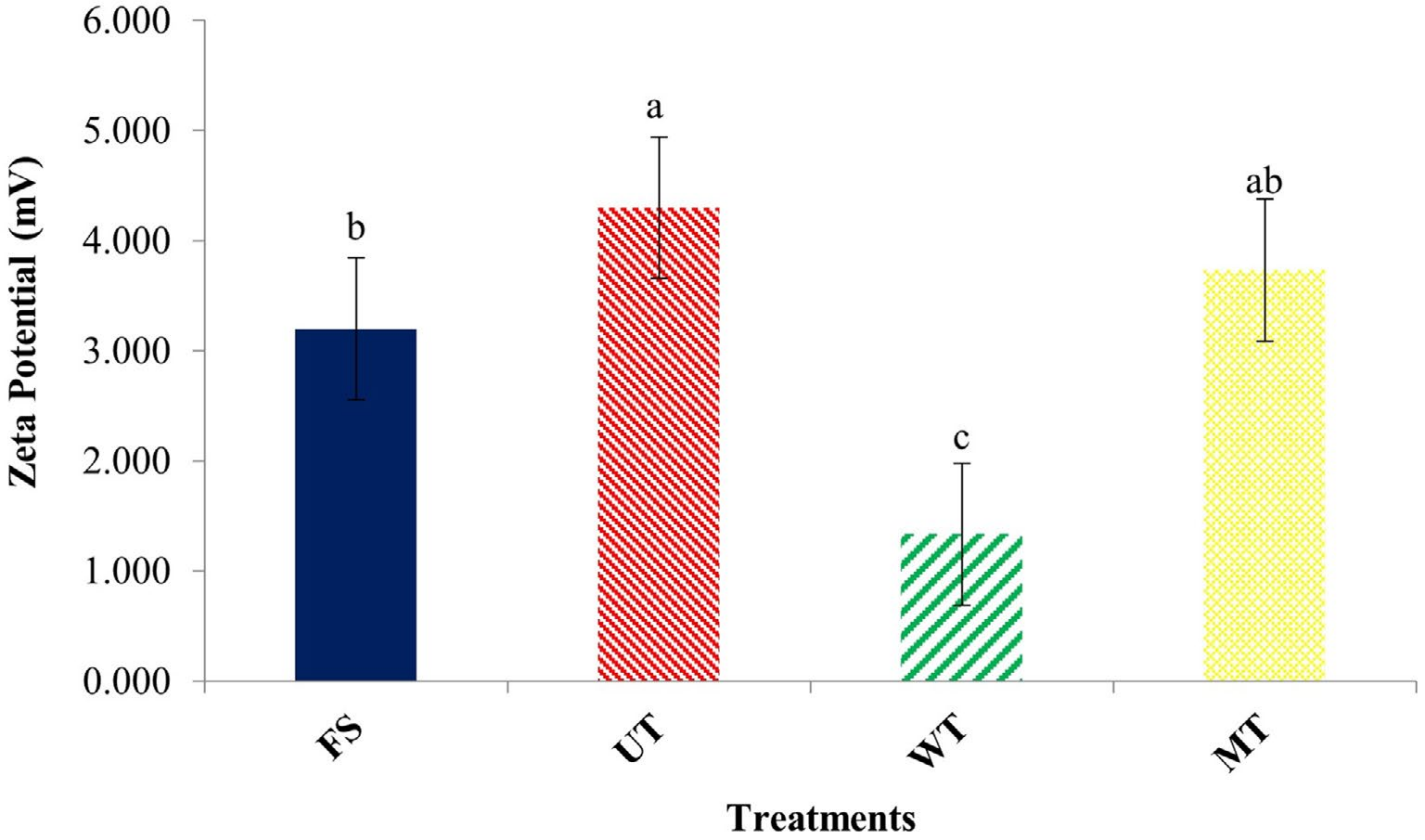
Zeta potential of myofibrillar proteins from common carp ( *Cyprinus carpio* ) fillets subjected to different thawing treatments. FS, fresh sample; MT, microwave thawing; UT, ultrasound thawing; WT, water immersion thawing.

Generally, a higher zeta potential indicates stronger electrostatic repulsion between suspended particles and enhanced colloidal stability (Esteban et al. 2016; Zhou and Yang 2020). As shown in Figure 5, zeta potential increased in both UT and MT treatments, with a significant improvement (*p* < 0.05) observed in the UT group. This effect may be attributed to ultrasound‐induced exposure of polar groups on the protein surface, leading to an increase in surface charge (Shi et al. 2019). These findings suggest that ultrasound‐treated proteins possessed the most uniform particle size distribution and the strongest interparticle repulsive forces, thereby inhibiting aggregation and enhancing protein stability (Wang et al. 2021).

#### 3.2.3 Turbidity

Turbidity, commonly measured by absorbance at 660 nm, serves as an important indicator of protein aggregation during heating (Ju and Kilara 1998). According to Wang et al. (2020), turbidity in thawed pork samples' increased significantly as the temperature rose from 30°C to 80°C. According to Ju and Kilara (1998), turbidity generally rises in parallel with the degree of protein aggregation. This may result from the unfolding of protein molecules and polymerization of monomeric proteins into larger aggregates (Ekezie et al. 2019).

**FIGURE 6:**
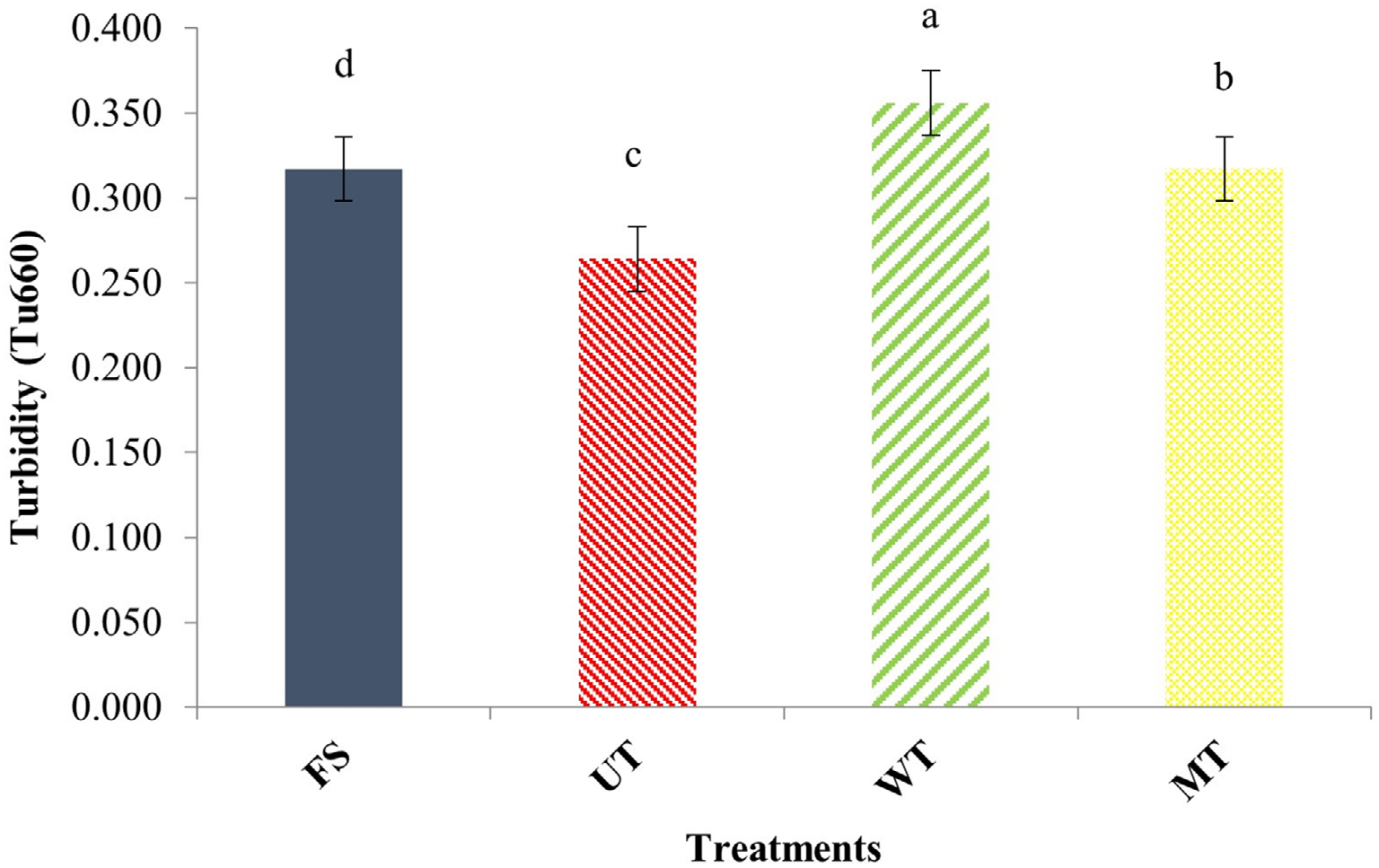
Turbidity of myofibrillar proteins from common carp ( *Cyprinus carpio* ) fillets subjected to different thawing treatments. FS, fresh sample; MT, microwave thawing; UT, ultrasound thawing; WT, water immersion thawing.

As shown in Figure 6, turbidity of thawed samples differed significantly from the control (*p* < 0.05). The water immersion‐thawed (WT) treatment showed the highest turbidity, differing significantly from other treatments (*p* < 0.05). According to Jia et al. (2015), the formation of larger or more numerous protein aggregates increases light scattering and reduces transmittance, thereby raising absorbance. Similar findings were reported by Wang et al. (2020) who reported higher turbidity in thawed pork samples, especially following water and microwave treatments, compared to controls. The ultrasound treatment showed the smallest deviation from the control, which aligns with the present results. The reduced turbidity observed in ultrasound‐treated samples can be attributed to cavitation‐induced shear forces, which disrupt protein aggregates into smaller particles.

## 4 Conclusion

This study provides a comprehensive evaluation of the impact of ultrasound (UT), water immersion (WT), and microwave (MT) thawing methods on the quality attributes of common carp ( *Cyprinus carpio* ) fillets and the structural properties of their myofibrillar proteins. The results demonstrate a distinct trade‐off between preserving protein integrity and maintaining macroscopic quality.

Ultrasound thawing (UT) proved superior for minimizing protein denaturation and aggregation, as evidenced by the smallest particle size (1460.80 nm), highest zeta potential, and lowest turbidity. This method was also most effective in maintaining the pH and minimizing cooking loss of the fillets. The improved performance of ultrasound‐assisted thawing can be explained by physical mechanisms associated with acoustic cavitation. The formation and collapse of microbubbles generate localized microstreaming and turbulence, which enhance heat and mass transfer around the sample surface. This process accelerates ice–water phase transition while reducing temperature gradients within the muscle tissue. As a result, heat diffusion becomes more uniform, limiting localized overheating and reducing protein denaturation and moisture migration. In contrast, water immersion thawing (WT) better preserved the visual and textural characteristics. It resulted in the lowest total color change (ΔE) and the highest values for hardness and gumminess among the thawed samples. Textural and microstructural (SEM) analyses confirmed that WT, along with MT, caused less physical damage to the muscle fibers compared to UT. Microwave thawing (MT) generally showed an intermediate performance but was notable for best retaining the springiness of the fillets. Crucially, SDS‐PAGE analysis confirmed that none of the thawing methods altered the primary structure of the myofibrillar proteins.

In conclusion, the choice of an optimal thawing method depends on the target quality parameter. UT is highly recommended for applications where protein functionality and water‐holding capacity are critical. WT is the preferable method when the priority is the preservation of color and textural hardness. These findings offer practical, science‐based guidance for the seafood industry to select thawing protocols aligned with specific product requirements. This study has some limitations such as small sample size, absence of oxidation analysis, lack of sensory evaluation, and laboratory‐scale conditions. Future studies should address these aspects and evaluate the scalability of ultrasound‐assisted thawing under industrial conditions.

## Author Contributions

**Abbas Zamani:** writing – review and editing, methodology, validation. **Seyed Vali Hosseini:** writing – review and editing, validation, resources. **Mina Esmaeili:** supervision, writing – review and editing, methodology, project administration, conceptualization, validation. **Mohammad Mehdi Asgari:** investigation, writing – original draft, project administration, formal analysis.

## Funding

This research received funding from the Vice Chancellor for Research and Technology, Sari Agricultural Sciences and Natural Resources University.

## Conflicts of Interest

The authors declare no conflicts of interest.

## Data Availability

The data that support the findings of this study are available from the corresponding author upon reasonable request.
